# Pharmacologic inhibition of somatostatin receptor 2 to restore glucagon counterregulation in diabetes

**DOI:** 10.3389/fphar.2023.1295639

**Published:** 2024-01-17

**Authors:** Emily G. Hoffman, Ninoschka C. D’Souza, Richard T. Liggins, Michael C. Riddell

**Affiliations:** ^1^ School of Kinesiology and Health Science, Muscle Health Research Centre, York University, Toronto, ON, Canada; ^2^ Zucara Therapeutics, Vancouver, BC, Canada

**Keywords:** hypoglycemia, type 1 diabetes, type 2 diabetes, glucagon, glucose counterregulation, somatostatin, somatostatin receptor 2 antagonist, pancreatic islets

## Abstract

Glucose homeostasis is primarily maintained by pancreatic hormones, insulin and glucagon, with an emerging role for a third islet hormone, somatostatin, in regulating insulin and glucagon responses. Under healthy conditions, somatostatin secreted from pancreatic islet δ-cells inhibits both insulin and glucagon release through somatostatin receptor- induced cAMP-mediated downregulation and paracrine inhibition of β- and α-cells, respectively. Since glucagon is the body’s most important anti-hypoglycemic hormone, and because glucagon counterregulation to hypoglycemia is lost in diabetes, the study of somatostatin biology has led to new investigational medications now in development that may help to restore glucagon counterregulation in type 1 diabetes. This review highlights the normal regulatory role of pancreatic somatostatin signaling in healthy islet function and how the inhibition of somatostatin receptor signaling in pancreatic α-cells may restore normal glucagon counterregulation in diabetes mellitus.

## 1 Introduction

The endocrine structure of the pancreas, called the islets of Langerhans, was first described over 150 years ago. The islets contain several hormone-secreting cell types that work as an integrated cellular network to regulate metabolism and maintain glucose homeostasis. Among these cells is the β-cell, which secretes insulin to lower blood glucose levels, the α-cell, which secretes glucagon to raise blood glucose levels, and the δ-cell, which secretes somatostatin (SST) to regulate insulin and glucagon secretion (Rahier et al., 1983). Over the past several decades, diabetes has become recognized as a complex, multi-hormonal disorder involving the three islet-cell types described above (Rorsman and Huising, 2018). In type 1 diabetes (T1D), insulin secretion is rapidly lost due to autoimmune-selective β-cell destruction (Siafarikas et al., 2012), whereas in type 2 diabetes (T2D), the delayed onset and gradual progression of insulin deficiency reflect exhausted β-cell efforts to compensate for insulin resistance (Weir et al., 2001). In T1D and late-stage (insulin deficient) T2D, the relationship between glucagon secretion and blood glucose levels is inverted, such that plasma glucagon levels rise after a mixed-meal and fall during hypoglycemia (Gaisano et al., 2012). Early evidence from diabetic rodent models suggests that the SST response to glucose may also be inverted in diabetes, with deficient SST release at high glucose (Hermansen, 1981; Abdel-Halim et al., 1993; van der Meulen et al., 2015) and excess release at low glucose (Vergari et al., 2020). Without restoring glucagon counterregulation to hypoglycemia in T1D and advanced T2D, strategies for improving glycemic control, including the use of newer insulin analogues (Lane et al., 2017; Wysham et al., 2017) and continuous glucose monitoring (Heinemann et al., 2018; Lin et al., 2019), fail to eliminate the risk of clinically significant hypoglycemia (Rickels, 2019). Accordingly, treatment-induced hypoglycemia remains the major barrier to optimal glycemic control in intensively-treated diabetes (Rickels, 2019). The use of novel somatostatin receptor 2 antagonists (SSTR2a) may resolve some of the dysfunction in glucagon counterregulation in diabetes, which could help to reduce the burden of treatment-induced hypoglycemia. This review compiles recent evidence for the role of SST receptor 2 (SSTR2) signaling in the pathophysiology of glucagon counterregulatory failure in diabetes. This review also summarizes the non-clinical and clinical data available to date on SSTR2 antagonism as a therapeutic approach to restoring glucagon counterregulation in diabetes.

## 2 Epidemiology of treatment-induced hypoglycemia in diabetes

Treatment-induced hypoglycemia is a pervasive clinical complication of intensive treatment with insulin analogues (T1D and T2D) and/or secretagogues (T2D) (Cryer, 2002). Annual rates of hypoglycemia are three to four times higher in adults with T1D than T2D (McCoy et al., 2012; Aronson et al., 2018), but owing to disease prevalence (∼20-fold that of T1D globally), T2D now accounts for the majority of severe diabetes-related hypoglycemic events (i.e., those resulting in hospitalization) (Heller et al., 2007; Ratner, 2018). Despite clinically significant improvements in glycemic control with the use of newer basal and bolus insulin analogues (Lane et al., 2017; Wysham et al., 2017) and glucose-sensing technologies (Heinemann et al., 2018; Lin et al., 2019), rates of severe hypoglycemia (resulting in emergency room visit or hospitalization) have increased (Lipska et al., 2014; Zhong et al., 2017). The use of insulin analogues across all age-groups in T2D, along with growing disease prevalence, likely account for these trends (Lipska et al., 2017). Further, the declining rate of all-cause mortality in diabetes, which was twice that of the general population between 1990 and 2010, has increased patient longevity, and thus, extended disease duration (Gregg, 2017; Ratner, 2018). Longer duration diabetes is a major risk factor for hypoglycemia (Weinstock et al., 2013) that is further compounded by advancing age (Berlin et al., 2005). Accordingly, hospital admission rates for hypoglycemia have surpassed admission rates for hyperglycemia/diabetic ketoacidosis in older adults living with diabetes in the US (Lipska et al., 2014; Zhong et al., 2017). Three large randomized controlled trials found that frequent, inadvertent hypoglycemia, secondary to intensive (HbA1C <7%) *versus* standard (HbA1C <9%) glycemic control in patients with longstanding T2D largely negated the cardiovascular benefits of intensive glycemic management (Patel et al., 2005; ADVANCE Collaborative Group et al., 2008; Duckworth et al., 2009). The recurrence of severe hypoglycemia in T2D is associated with increased risk of adverse vascular outcomes (Patel et al., 2005), arrhythmias (Chow et al., 2014), and early dementia (Whitmer et al., 2009). In both T1D and T2D, one or more severe hypoglycemic events over 5 years raises mortality rates ∼3.5-fold (Beck et al., 2019). To offset this risk, clinical practice guidelines recommend a temporary relaxation in glycemic targets upon self-report of one or more severe hypoglycemic events (ADAPP, 2022), which typically precludes the maintenance of normal HbA1C levels in adults living with diabetes (Zammitt and Frier, 2005). As a result, glycemic control in diabetes is largely limited by the trade-off between long-term vascular protection and short-term hypoglycemia avoidance (Peacey et al., 2000).

## 3 Glucagon counterregulatory failure in diabetes

Glucose counterregulation during hypoglycemia is mediated by a hierarchy of redundant neuro-hormonal responses in healthy individuals. First, a drop in blood glucose levels within the euglycemic range causes insulin secretion to switch-off at a glucose threshold of ∼4.4–4.7 mmol/L (Mitrakou et al., 1991). The ratio of insulin to glucagon in the hepatic portal vein (pancreatic effluent) dictates glucose mobilization from the liver. Therefore, lower portal insulin levels favor higher glucose output, as well as lower glucose uptake by insulin-sensitive tissues (excluding the brain) (Cryer et al., 1984). A drop in blood glucose levels below the euglycemic range (≤3.9 mmol/L, classified as level 1 hypoglycemia) (International Hypoglycaemia Study Group, 2017) activates the release of chief counterregulatory hormone, glucagon, from islet α-cells at a glucose threshold of 3.6–3.9 mmol/L (Mitrakou et al., 1991) Along with epinephrine, glucagon stimulates hepatic glycogenolysis and gluconeogenesis to prevent and correct hypoglycemia (Cryer et al., 1984). Autonomic and neuroglycopenic symptoms typically begin to appear during level 2 hypoglycemia (<3.0 mmol/L) (International Hypoglycaemia Study Group, 2017) followed by the onset of cognitive impairment (<2.8 mmol/L) (Mitrakou et al., 1991). Level 3 hypoglycemia denotes severe cognitive impairment requiring external assistance for glycemic recovery (International Hypoglycaemia Study Group, 2017).

The incidence of biochemical hypoglycemia (defined here as a whole blood glucose concentration ≤3.9 mmol/L) in people without diabetes is low, but can occur in the post-prandial (fed) state as a result of endogenous hyperinsulinism (e.g., insulinomas), alcohol consumption, and certain medications (e.g., lithium, angiotensin-converting enzyme inhibitors, angiotensin receptor blockers, and non-selective β-blockers) or in the post-absorptive (fasted) state as a result of prolonged exercise, bariatric surgery, severe sepsis, malnutrition (e.g., anorexia nervosa), and renal, hepatic, or cardiac failure (Cryer et al., 2009). In a setting of T1D and late-stage (insulin-deficient) T2D, hypoglycemia is the most common complication of intensive glucose-lowering therapy using insulin analogues and/or secretagogues (i.e., sulfonylureas and glinides; T2D only) (Cryer, 2002). Hypoglycemia risk stems from imperfect (non-physiologic) insulin replacement in a setting of defective glucose counterregulation (Cryer, 2002). Since insulin analogues and secretagogues do not act in a glucose-sensitive fashion, they cannot respond to dynamic changes in insulin requirement. As a result, imperfect dosing can lead to a state of relative hyperinsulinemia (i.e., excess systemic insulin levels relative to blood glucose concentration), in which glucose counterregulatory mechanisms become critical to protecting against hypoglycemia (Rahier et al., 1983). However, α-cells fail to meet this demand, becoming increasingly “blind” (unresponsive) to hypoglycemia with longer duration diabetes and progressive insulin deficiency (Madsbad et al., 1982; Cryer, 2005; Siafarikas et al., 2012; Zenz et al., 2018). Hypoglycemia in the range of ∼2.5–3.5 mmol/L typically fails to trigger a clinically meaningful increase in plasma glucagon levels (systemic values fail to rise above ∼60 pg/mL when the expected increase is >100 pg/mL) within months of T1D diagnosis (Siafarikas et al., 2012), thereby shifting the counterregulatory burden to sympathoadrenal (epinephrine) and other autonomic mechanisms (Cryer, 2005). However, these neuroendocrine pathways are easily overwhelmed by the use of insulin analogues and/or secretagogues, leading to inadvertent hypoglycemia and the clinical syndrome of hypoglycemia-associated autonomic failure (HAAF) (Cryer, 2005). In short, recent antecedent hypoglycemia lowers glycemic thresholds for sympathoadrenal activation during subsequent hypoglycemia, resulting in diminished counterregulatory and neurogenic symptom responses (Cryer, 2005). The natural history of α-cell “blindness” is less clear in T2D but is thought to mirror the progression of endogenous insulin deficiency, which is much slower in T2D than T1D (Segel et al., 2002; Zammitt and Frier, 2005). The gradual depletion of functional β-cells in T2D and resulting impairment to the glucose-sensing capacity of neighboring α-cells, may help to explain why treatment-induced hypoglycemia and HAAF become limiting to glycemic control with longer disease duration (Segel et al., 2002; Zammitt and Frier, 2005).

### 3.1 Pancreatic SST and its regulation

**FIGURE 1:**
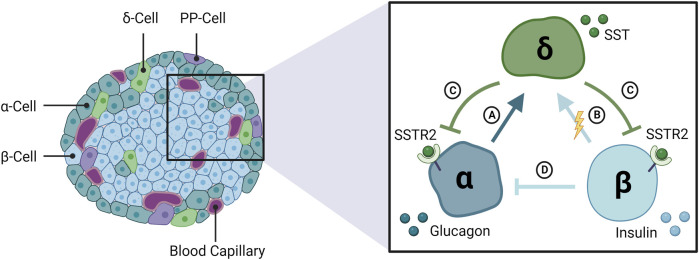
The interrelationship among α-, β-, and δ-cells of the endocrine pancreas. The pancreatic islets of Langerhans are composed of three glucose-regulating cell types, α- β- and δ-cells, that secrete glucagon, insulin, and somatostatin (SST), respectively. SST secretion is stimulated by **(A)** glucagon and **(B)** β-cells, likely via electrical coupling with δ-cells rather than secreted insulin. **(C)** SST feeds back to inhibit glucagon and insulin release via SSTR2 in human islets. **(D)** Glucagon secretion is inhibited by insulin and other β-cell secretory products. SSTR2: somatostatin receptor 2; PP: pancreatic polypeptide.

SST-14 is an inhibitory peptide hormone secreted by pancreatic δ-cells, which constitute ∼5–10% of the total islet-cell mass (Figure 1) (Folli et al., 2018). Circulating levels of SST are unaffected by pancreatectomy in animals (Marre et al., 1983; Taborsky and Ensinck, 1984) and humans (Gutniak et al., 1987), suggesting a negligible contribution by the pancreas to plasma SST. The predominant isoform in circulation, SST-28, originates from enteroendocrine cells of the gastrointestinal (GI) tract where it regulates digestive functions (i.e., reduces gastric and intestinal motility) by inhibiting the release of several GI hormones (Francis et al., 1990). Both SST isoforms bind to a family of five SSTR subtypes (SSTR1-5) distributed throughout the brain and neuroendocrine tissues (Raulf et al., 1994). Islet-derived SST is most concentrated in the portal vein (pancreatic effluent), though detection is hampered by the hormone’s short half-life in circulation (<1 min) (Rorsman and Huising, 2018) and a lack of commercially available assays that can select for either biological isoform. Consequently, islet secretion of somatostatin cannot be reliably measured from plasma sampling *in vivo* and must instead be measured from the *in situ* perfused pancreas or from *in vitro* preparations of isolated islets or dispersed δ-cells.

**FIGURE 2:**
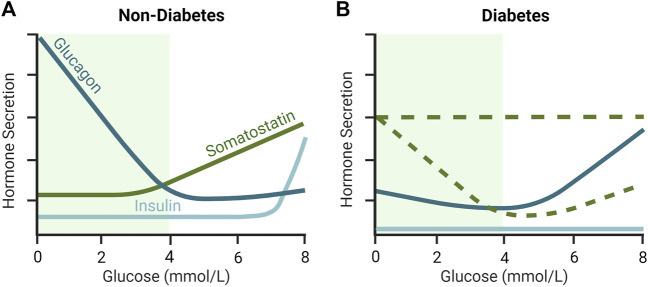
Glucose-dependent secretion of insulin, glucagon and somatostatin (SST) in **(A)** non-diabetic and diabetic **(B)** islets. In non-diabetic rodent islets, glucagon secretion is maximally stimulated at low glucose and declines to a nadir at an islet glucose concentration of ∼4–5 mmol/L. SST secretion is low during hypoglycemia and increases as glucose rises above 3 mmol/L. In contrast, in diabetes, counterregulatory glucagon secretion is supressed during hypoglycemia and SST is elevated. The loss of β-cell mass in T1D and late-stage T2D rodent islets results in low insulin secretion, but also increased SST secretion, at least during hypoglycemia, and elevations in glucagon secretion in euglycemia and hyperglycemia. As represented by the dashed green lines, the glucose-dependent secretion of SST in diabetes in not completely understood and different animal models provide conflicting views. More specifically, it is unclear whether SST secretion remains relatively stable (i.e., unresponsive) with increasing glucose concentration, or whether it drops below non-diabetic levels during hyperglycemia in diabetes. Green shaded region indicates the glycemic range over which the majority of counterregulatory glucagon secretion occurs in non-diabetic islets. Inspired by Huising et al. (2018); Noguchi and Huising (2019).

δ-cells are active throughout the physiologic glucose range, reflecting the important “gatekeeping” role of SST within the islets (Rorsman and Huising, 2018). In isolated islets, stimulated SST secretion is initiated at glucose concentrations as low as 3 mmol/L and increases dose-dependently toward 20 mmol/L glucose, with half maximal stimulation at ∼5–6 mmol/L glucose in rodent islets (Vieira et al., 2007) (Figure 2A) and ∼10 mmol/L glucose in human islets (Walker et al., 2011). Between 1 and 20 mmol/L glucose, SST secretion increased >10-fold in mouse islets (Vieira et al., 2007; Vergari et al., 2020) and ∼3–3.5-fold in human islets (Ramracheya et al., 2010; Walker et al., 2011; Vergari et al., 2020) between 1 and 20 mmol/L glucose, indicating a larger magnitude response in mice. SST release is stimulated by β-cell factors, γ-Aminobutyric acid (GABA) (Braun et al., 2010) and urocortin 3 (UCN3) (van der Meulen et al., 2015), that are co-secreted with insulin in response to glucose. UCN3 accounts for the majority of SST secretion during hyperglycemia and represents a form of autoregulation by the β-cell that feeds back (on a brief delay) via SST to inhibit insulin secretion by paracrine effect (Svendsen and Holst, 2021). However, secreted β-cell signals appear less important for the regulation of δ-cell activity under hypoglycemic conditions (Svendsen and Holst, 2021). Speculation that SST secretion is mediated by glucose alone below the threshold for stimulated insulin secretion (∼7 mmol/L; Figure 2A) (Svendsen and Holst, 2021) has been challenged by the discovery of electrical coupling between β- and δ-cells (Briant et al., 2018; Miranda et al., 2022). Islet cells are electrically excitable cells that secrete hormones in response to membrane depolarization (Briant et al., 2018). Mathematical modeling of δ-cells suggests that hyperpolarizing (inhibitory) membrane currents spread from β-cells to δ-cells via gap junction connections under low glucose conditions (≤3.9 mmol/L), in turn, suppressing δ-cell activity and SST release (Briant et al., 2018; Miranda et al., 2022). In other words, δ-cells are normally electrically silenced by neighboring β-cells at low glucose, independent of diffusible paracrine factors (Briant et al., 2018; Miranda et al., 2022). The removal of inhibitory SST signals from the paracrine environment at low glucose may favor the activation of neighboring α-cells and consequent glucagon release (Briant et al., 2018; Miranda et al., 2022).

### 3.2 Paracrine regulation of insulin and glucagon secretion by SST

Islet-derived SST inhibits insulin and glucagon secretion by activating SST receptors in β- and α-cells, respectively. Both cell types have been co-localized with four (SSTR1-3 and SSTR5) of five (SSTR1-5) known SSTR subtypes in humans, with preferential expression of SSTR1 and SSTR5 by β-cells and SSTR2 by α-cells (Kumar et al., 1999). Yet, SSTR expression does not necessarily reflect the subtype-specific regulation of individual pancreatic hormones (Singh et al., 2007; Kailey et al., 2012). Selective agonism of all five receptors has revealed SSTR2 as the functionally dominant SSTR in human α- and β-cells, with lesser contributions by SSTR1 and SSTR5 to the regulation of both hormones (Singh et al., 2007; Kailey et al., 2012). In rodents, insulin and glucagon are inhibited by SSTR2 and SSTR5, respectively (Strowski et al., 2000). Therefore, insulin and glucagon secretion are regulated by a common SSTR (SSTR2) in humans (Kailey et al., 2012) and by distinct SSTR subtypes (SSTR5 for insulin and SSTR2 for glucagon) in rodents (Strowski et al., 2000).

The uptake and metabolism of glucose drives ATP production by both α- and β-cells, leading to the closure of ATP-sensitive potassium (K_ATP_) channels. In β-cells, the resulting depolarization by intracellular potassium accumulation triggers action potential firing and a rise in intracellular calcium that stimulates insulin exocytosis (Rorsman and Ashcroft, 2018). In α-cells, moderate K_ATP_ channel activity at low glucose concentrations establishes a resting membrane potential that drives conductance through voltage-gated calcium (T- and L-type) and sodium channels. The resulting depolarization triggers action potential firing that opens voltage-gated calcium channels. In turn, the accumulation of intracellular calcium stimulates glucagon exocytosis (Briant et al., 2016).

SSTR signaling suppresses hormone secretion from α- and β-cells through four main effector pathways: 1) inactivation of inhibitory G_αi_-coupled proteins, which decreases adenylate cyclase activity and cytoplasmic levels of cAMP (Braun, 2014; Elliott et al., 2015), 2) activation of sodium-potassium pumps (β-cells only) (Dickerson et al., 2022) and G protein-gated inwardly rectifying potassium channels, which hyperpolarizes the plasma membrane and inhibits action potential firing (Kailey et al., 2012; Hartig and Cox, 2020), 3) inactivation of voltage-gated calcium channels, which reduces depolarization-induced calcium influx (Hsu et al., 1991; Kailey et al., 2012), and 4) direct inactivation of hormone exocytosis, downstream of calcium signaling, in a cAMP-independent manner (Kailey et al., 2012).

### 3.3 Physiologic regulation of counterregulatory glucagon secretion

The regulation of glucagon secretion from pancreatic α-cells relies on a complex interplay of humoral (circulating nutrients, hormones, and neurotransmitters), neural (sympathetic, parasympathetic, and sympathoadrenal), autocrine, and paracrine inputs (Walker et al., 2011). However, the preservation of physiologic glucagon secretion from intact islets *in vitro* (Omar-Hmeadi et al., 2020) suggests a dominant contribution by intra-islet factors (Gilon, 2020). As illustrated in Figure 2A, glucagon secretion from healthy islets is inhibited by glucose in a concentration-dependent manner with maximal effect at 4–5 mmol/L (MacDonald et al., 2007; Vieira et al., 2007). The onset of glucose-stimulated insulin secretion at ∼7 mmol/L (Figure 2A) discounts a role for insulin or co-secreted β-cell factors in the regulation of glucagon secretion below 7 mmol/L glucose (Walker et al., 2011). Alternatively, the marked stimulation of counterregulatory glucagon secretion following SST knockout (Hauge-Evans et al., 2009) or immunoneutralization (de Heer et al., 2008), or pharmacological inhibition of SSTR2 (Xu et al., 2020; Svendsen and Holst, 2021), suggests tonic inhibition of the α-cell by SST. In agreement, SSTR2 antagonism stimulated glucagon secretion ∼1.8-fold at 3.5 mmol/L glucose *versus* ∼4.5-fold at 12 mmol/L glucose in the perfused healthy mouse pancreas (Svendsen and Holst, 2021). These findings suggest that SST is important for inhibiting glucagon secretion at higher glucose concentrations (>6 mmol/L) and that its removal under low glucose conditions (<3 mmol/L) releases an inhibitory “brake” on counterregulatory glucagon secretion.

### 3.4 Pathologic regulation of glucagon counterregulation in diabetes

The paracrine basis of defective glucagon counterregulation in T1D and advanced (insulin-deficient) T2D has eluded islet physiologists for over 40 years. That said, increased islet content (*ex vivo*) (Orci et al., 1976; Baetens et al., 1978; Abdel-Halim et al., 1993) and secretion (*in situ* and *in vitro*) (Hermansen et al., 1979; Weir et al., 1981; Abdel-Halim et al., 1993) of SST under low glucose conditions have long implicated SST in the pathogenesis of defective glucagon counterregulation in animal models of diabetes. More recently, Vergari et al. measured glucose-dependent SST secretion in the islets of hyperglycemic mice lacking the Krebs cycle enzyme fumarate hydratase in pancreatic β cells (Fh1βKO), a model of progressive insulin deficiency reminiscent of T2D (Adam et al., 2017; Vergari et al., 2020). As illustrated in Figure 2B, SST secretion was increased 6-fold in Fh1βKO islets relative to control mouse islets at 1 mmol/L glucose, which correlated with a >75% reduction in glucagon secretion (Vergari et al., 2020). Consistent with the observation of elevated SST secretion at 1 mmol/L glucose, application of a SST receptor 2 antagonist (SSTR2a, CYN154806) in Fh1βKO islets stimulated glucagon release by 143% ± 11% compared to 13% ± 14% in control mouse islets (Vergari et al., 2020).

In intact isolated islets from humans with T2D, glucagon secretion at 1 mmol/L glucose was lower (65% on average) than in non-diabetic human islets, though, a reciprocal trend towards elevated SST secretion failed to reach statistical significance as it did in Fh1βKO mouse islets (Vergari et al., 2020). The SSTR2a CYN154806, stimulated glucagon secretion at 1 mmol/L glucose but only in islets (2 of 3) with lower glucagon secretion relative to controls (Vergari et al., 2020). While these preliminary findings must be confirmed in larger samples of T2D islets, they support the conclusions that 1) suppression of SST secretion from islet δ-cells acts as a permissive signal for counterregulatory glucagon release, and 2) this permissive signal may be compromised in T1D and advanced T2D (Vergari et al., 2020). As mentioned above, islet δ-cells are electrically silenced by neighboring β-cells via gap junction connections at low glucose (Briant et al., 2018; Miranda et al., 2022). Release of this hyperpolarizing ‘brake’ in conditions of β-cell death, like diabetes, is expected to increase the electrical excitability of the δ-cell and resulting SST output above normal (Briant et al., 2018; Miranda et al., 2022; Singh et al., 2022). In support of this theory, δ-cell dispersion (i.e., removal of β-cell coupling) may stimulate δ-cell hyperactivity at low glucose concentrations (Berts et al., 1996). While this review focuses on SST-dependent mechanisms of glucagon counterregulatory failure in insulin-deficient diabetes, other causative mechanisms, such as changes in the anatomical and paracrine relationships between α- and β-cells, may also play a role (Weir and Bonner-Weir, 2023).

## 4 Treatment with SSTR2a for hypoglycemia prevention in diabetes

**FIGURE 3:**
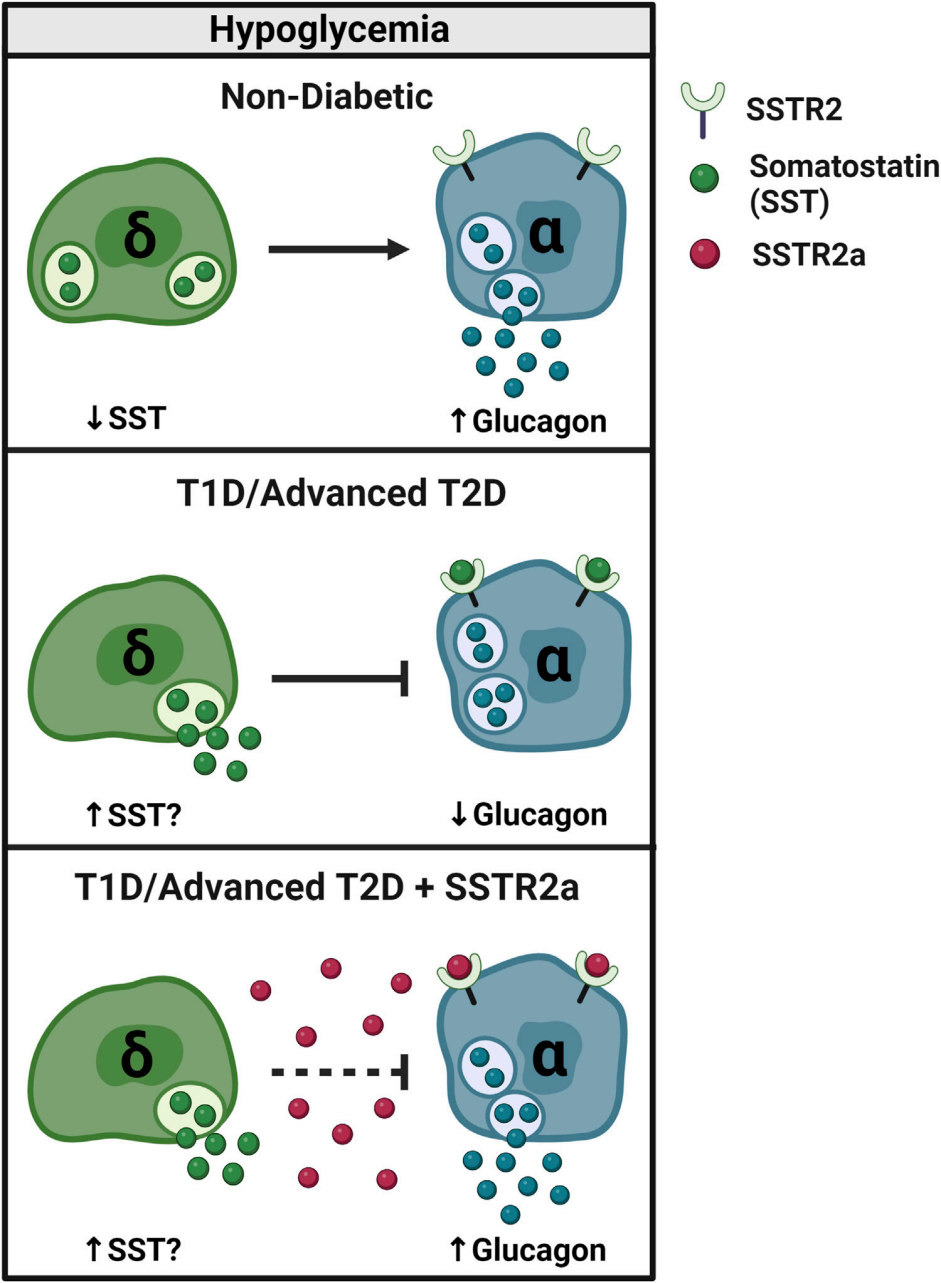
SSTR2 antagonism reverses glucagon counterregulatory failure in T1D and advanced T2D. In non-diabetic conditions, SST secretion decreases and glucagon secretion increases during hypoglycemia (≤3.9 mmol/L) to restore euglycemia. In T1D, counterregulatory glucagon secretion is impaired, possibly due to an increase in inhibitory SST signaling. By blocking SST signaling to the α-cell, pharmacologic SSTR2a improve the glucagon response to insulin-induced hypoglycemia in rodents and humans with T1D and potentially advanced (insulin-deficient) T2D.

Figure 3 depicts α- and δ-cell responses to hypoglycemia in the non-diabetic and insulin-deficient diabetic states. As discussed in section 3, SST inhibits glucagon secretion via SSTR2 expressed by islet α-cells. In the non-diabetic state, SST secretion and resultant SSTR2 activation are low during hypoglycemia, allowing for counterregulatory glucagon release. In conditions of T1D and late-stage T2D, a pathological elevation in SST secretion during hypoglycemia may underscore the acquired defect in counterregulatory glucagon release. By antagonizing this upregulated signaling pathway in diabetes, SSTR2a’s release an inhibitory brake on endogenous glucagon secretion during insulin-induced hypoglycemia.

### 4.1 Effects of SSTR2a in rodent models of T1D

As an adjunct to intensive glucose lowering treatment with insulin analogues and/or secretagogues (i.e., sulfonylureas and glinides), agents that block SSTR2 signaling in pancreatic α-cells may help alleviate the risk of treatment-induced hypoglycemia in T1D and late-stage T2D. The therapeutic effect of a selective SSTR2a, PRL-2903, on glucagon counterregulation was first demonstrated in streptozotocin (STZ) and biobreeding models of T1D during acute (Yue et al., 2012; Karimian et al., 2013) and recurrent (Yue et al., 2013) hypoglycemia. Normalization of plasma glucagon levels with PRL-2903 fully alleviated the dependence on glucose infusion for the maintenance of clamped hypoglycemia in biobreeding rats (Karimian et al., 2013) and reduced exogenous glucose dependence in STZ rats when hypoglycemia was induced with low- (5 U/kg) but not high- (10 U/kg) dose insulin (Yue et al., 2012). Further, no drug effect was observed under basal conditions (Yue et al., 2012). In recurrently hypoglycemic STZ-T1D rats, PRL-2903 prevented a 20-fold reduction in the plasma glucagon response to recurrent hypoglycemia after 5 antecedent episodes in 3 days (Yue et al., 2013). SSTR2 blockade also raised the glucose nadir from level 3 (2.7 mmol/L) to level 1 (3.7 mmol/L) hypoglycemia and reduced time spent in hypoglycemia (≤3.9 mmol/L) by ∼50 min compared to vehicle treatment (Yue et al., 2013).

While this review is focused mainly on treatment-induced hypoglycemia, the contribution of physical activity to hypoglycemia risk in T1D cannot be overlooked. Aerobic forms of activity (e.g., walking, cycling, endurance exercise) often result in immediate and/or delayed episodes of hypoglycemia in people with T1D. This risk may stem from lower baseline hepatic glycogen stores [particularly if glycemic control is suboptimal (Bischof et al., 2002)], impaired or absent glucagon release during exercise (McCarthy et al., 2022), and increased muscle glucose disposal without a physiologic reduction in circulating insulin levels (i.e., relative hyperinsulinemia) (Mallad et al., 2015). These metabolic disturbances collectively result in a blunting of hepatic glucose production during exercise. To help restore glucagon counterregulation and limit hypoglycemia during exercise, Leclair et al. examined the efficacy of SSTR2 antagonism in STZ-T1D rodents during combined bolus insulin/exercise challenges (Leclair et al., 2016). For this, STZ-T1D rodents performed 30 min of treadmill exercise in a hyperinsulinemic state with or without PRL-2903 (10 mg/kg, IP) given 90 min before exercise (Leclair et al., 2016). Plasma glucagon levels in the SSTR2a group rose more than 2-fold from baseline by the end of exercise, without a detectable glucagon response in the saline group (Leclair et al., 2016). When the hyperinsulinemic exercise challenge was repeated on a subsequent day, PRL-2903 raised glucagon levels 3.5-fold in animals previously treated with the antagonist (Leclair et al., 2016). During a third exercise challenge, animals previously treated with saline showed improved glucagon counterregulation when given PRL-2903 for the first time (Leclair et al., 2016). Notably, PRL-2903 only augmented plasma glucagon levels during exercise- and insulin-induced hypoglycemia, suggesting a glucose-dependent drug effect (Leclair et al., 2016). These findings reveal that SSTR2 antagonism may help to improve glucagon counterregulation in animals exposed to recurrent exercise-induced hypoglycemia.

Since then, Farhat and others have demonstrated the superior efficacy of more potent SSTR2a peptide, ZT-01 (Zucara Therapeutics), compared to PRL-2903, in a setting of insulin-induced hypoglycemia (Farhat et al., 2022). ZT-01 raised the peak plasma glucagon response ∼4-fold over PRL-2903 when compared head-to-head during clamped hypoglycemia in STZ-T1D rats. ZT-01 was also more effective than PRL-2903 at raising the blood glucose nadir in free-living STZ-T1D rats after bolus insulin challenge, thereby reducing the incidence of hypoglycemia and time spent in the hypoglycemic range (levels 1 and 2) (Farhat et al., 2022). Additionally, the pharmacokinetic properties of ZT-01 were more favorable than PRL-2903 when delivered by intraperitoneal (IP) or subcutaneous (SC) route in STZ-T1D rats (Farhat et al., 2022). Namely, cumulative drug exposure (based on AUC of plasma drug concentration) was 11 times higher for ZT-01 than PRL-2903 over a 24-h period (Farhat et al., 2022). After establishing the superior therapeutic and pharmacokinetic properties of ZT-01 as compared to PRL-2903, the authors identified the minimum effective dose level of ZT-01 (0.3 mg/kg) that could increase glucagon secretion during clamped hypoglycemia (∼2.5 mmol/L) in poorly controlled, insulin-treated STZ-T1D rats (Farhat et al., 2022). Based on these and other safety and efficacy trials, ZT-01 has recently advanced to clinical trials in adults with (Abitbol et al., 2023) and without T1D (NCT05007977, 2023). Drug safety and pharmacodynamics were assessed in phase 1 trials (Abitbol et al., 2023; NCT05007977, 2023) and a phase 2 study is currently recruiting participants with T1D to assess the efficacy of ZT-01 in reducing nocturnal hypoglycemia (NCT05762107, 2023). In the phase 1b trial (manuscript not yet peer-reviewed), the peak plasma glucagon response to ZT-01 (3 or 20 mg) was evaluated in subjects with T1D during clamped level 1 (3.5 ± 0.3 mmol/L) and level 2 (2.6 ± 0.2 mmol/L) hypoglycemia (Abitbol et al., 2023). During level 1 hypoglycemia, ZT-01 raised peak plasma glucagon levels by up to 15 pg/mL from baseline, whereas no response was observed with placebo (Abitbol et al., 2023). During level 2 hypoglycemia, the placebo response was 8 pg/mL, and the increase from baseline to peak glucagon level was >3-fold higher with ZT-01 than with placebo (Abitbol et al., 2023).

### 4.2 Effects of SSTR2a in a non-diabetic rodent model of recurrent hypoglycemia

Each episode of insulin-induced hypoglycemia blunts the counterregulatory glucagon response to subsequent hypoglycemia in healthy and diabetic rodents (Hoffman et al., 2021; Yue et al., 2013) and humans (Heller and Cryer, 1991; Davis et al., 2009). Unlike epinephrine, defective glucagon counterregulation, which develops in parallel with HAAF, is not corrected by short-term hypoglycemia avoidance in humans with T1D (Liu et al., 1996; Flatt et al., 2023). To determine whether this glucagon secretory defect is mediated (at least in part) by SST signaling, and whether it responds to SSTR2a treatment, we tested PRL-2903 in healthy male rats subjected to three antecedent episodes of insulin-induced hypoglycemia (Hoffman et al., 2021). The absence of diabetes in this rodent model allowed us to isolate and reverse the glucagon secretory defect resulting from antecedent hypoglycemia *per se* (independent of diabetes-related defects) (Hoffman et al., 2021). In this ‘hypoglycemia conditioned’ model, the SSTR2a, PRL-2903, reversed the cumulative deficit (68%) in counterregulatory glucagon levels resulting from three antecedent episodes of hypoglycemia (Hoffman et al., 2021). Recovery of the glucagon response was associated with a 25 min delay in the onset of subsequent (day 4) hypoglycemia and accelerated hepatic glycogenolysis, as assessed by liver glycogen content (Hoffman et al., 2021). By restoring glucagon counterregulation in a setting of recurrent hypoglycemia, SSTR2a may have promising therapeutic implications for managing hypoglycemia risk in people with HAAF (Hoffman et al., 2021). Not only would treatment provide temporary avoidance of hypoglycemia without compromising metabolic control, it may help to prevent the recurrence of HAAF once intensive insulin therapy is resumed.

### 4.3 Effects of SSTR2a in rodent models of pre-diabetes

The SSTR2a, CYN154806, was examined in the perfused pancreas and isolated islets of normoglycemic high-fat-fed (HFF) mice (Kellard et al., 2020). Unlike reports of impaired glucagon secretion at low glucose in T1D and T2D islets, glucagon output from HFF islets was 2-fold higher than control islets at 1 mmol/L glucose (Kellard et al., 2020). The observed hypersecretion of glucagon at low glucose was attributed to a 30% reduction in SST secretion and acquired α-cell resistance to SST signaling that could not be explained by changes in α-cell expression of SSTR2 (Kellard et al., 2020). Accordingly, SSTR2 antagonism at 6 mmol/L glucose had little to no effect on glucagon secretion in HFF islets, unlike the marked stimulatory effect observed in control islets. SSTR2 antagonism was not tested at lower glucose concentrations in this model and remains a goal of future studies (Kellard et al., 2020).

The effects of a SSTR2a, ZT-01, have also been investigated in a rat model of HFF/STZ-induced pre-diabetes (Hoffman et al., 2023). The combined presentation of basal hyperinsulinemia and mild hyperglycemia in this model suggests inadequate β-cell compensation for insulin resistance (i.e., relative insulin deficiency), and therefore, recapitulates a more advanced pre-diabetic phenotype than high-fat feeding alone (Hoffman et al., 2023). Plasma analysis revealed a mild attenuation in the magnitude (glucagon AUC) and responsiveness (increase in plasma glucagon concentration per 1 mmol/L drop in blood glucose concentration) of glucagon to bolus insulin challenge in this pre-diabetic model (Hoffman et al., 2023). Pretreatment with the selective SSTR2a, ZT-01, augmented the plasma glucagon response during hypoglycemia by all measures (peak concentration, AUC, and responsiveness) in both healthy and pre-diabetic rats (Hoffman et al., 2023). Blood glucose was elevated above control levels within 30 min of ZT-01 treatment in pre-diabetic rats, and remained elevated for 30 min after hypoglycemia induction (Hoffman et al., 2023). Further, ZT-01 delayed hypoglycemia onset by 15 min compared to vehicle treatment in pre-diabetic but not healthy rats (Hoffman et al., 2023). Consistent with evidence from SST knockout islets (Hauge-Evans et al., 2009), the C-peptide “switch-off” response to insulin-induced hypoglycemia was unaffected by ZT-01 treatment in healthy and in pre-diabetic rats (Hoffman et al., 2023). This finding confirmed antagonist selectivity for SSTR2 (expressed by islet α-cells) over SSTR5 (expressed by rodent β-cells) (Strowski et al., 2000; Hoffman et al., 2023). However, the glucagon-stimulating effect of ZT-01 was not dependent on low-glucose conditions in healthy or pre-diabetic rats, which may aggravate basal hyperglucagonemia and/or hyperglycemia at the dose evaluated in pre-diabetes (Hoffman et al., 2023) (which is three to ten times higher than the minimum effective dose in T1D rats) (Farhat et al., 2022). The data further suggest that α-cells may begin to develop “glucose blindness” early on in the course of β-cell failure (Hoffman et al., 2023). Accordingly, early intervention with SSTR2a may offer a means of preventing, rather than simply reversing, a functional α-cell deficit in T2D.

### 4.4 Effects of SSTR2a in a rodent model of T2D

Only one published abstract to date has profiled the potential *in vivo* effects of single-dose-therapy with a SSTR2a on glucose counterregulation in T2D (manuscript in peer-review). The rat model of HFF/low-dose (35 mg/kg) STZ used in this study was severely hyperglycemic (∼20 mmol/L), with a 50% reduction in basal C-peptide levels and deficient glucagon counterregulation relative to HFF controls (D’Souza et al., 2023a). When given 60 min before bolus insulin overdose, the SSTR2a, ZT-01 (3 mg/kg, SC), raised peak plasma glucagon levels ∼2-fold compared to vehicle treatment (D’Souza et al., 2023a). Further, pre-treatment with ZT-01 reduced the incidence of hypoglycemia by 40% and delayed the onset of hypoglycemia in affected animals by ∼80 min compared to vehicle (D’Souza et al., 2023a). Notably, ZT-01 did not appear to exacerbate hyperglycemia, at least when administered at meal time in the form of an oral glucose tolerance test (OGTT; 2 g/kg D-glucose), thereby suggesting a glucose-dependent drug effect in this model (D’Souza et al., 2023b). These preliminary non-clinical data suggest that SSTR2a may have therapeutic implications for hypoglycemia prevention in late stage (insulin-deficient) T2D (D’Souza et al., 2023a).

## 5 Sustained treatment with SSTR2a for hypoglycemia prevention in diabetes

The effects of sustained SSTR2 antagonism on overall glycemia and hypoglycemia prevention in diabetes are unclear. Sustained delivery of peptide antagonists may be facilitated by longer half-lives, improved drug formulations, infusion pump technology, and/or microneedle (MN) patches (GhavamiNejad et al., 2022; Aleali et al., 2023). In one study, Wu et al. developed a MN patch using methacrylated hyaluronic acid (MeHA) for transdermal delivery of PRL-2903 in STZ-T1D rats (GhavamiNejad et al., 2022). When applied 30 min before bolus insulin overdose (2–3 U/kg), the PRL-2903-loaded MN patch augmented plasma glucagon levels and resisted the onset of hypoglycemia for up to 75 min in STZ-T1D rats compared to sham-patch controls (GhavamiNejad et al., 2022). Further, the results of a molecular dynamics simulation suggested that MEHA may stabilize the structure of native PRL-2903 by protecting it from thermal and UV damage that reduces its biological activity (GhavamiNejad et al., 2022). These findings suggest that MN patch technology may be a convenient and effective system for sustained delivery of SSTR2a peptides (GhavamiNejad et al., 2022); however, assessment of patch compatibility with PRL-2903 analogue, ZT-01, will help determine its candidacy for clinical use.

In another recently published abstract, low-dose ZT-01 was delivered by subcutaneously-implanted mini-osmotic pumps in HFF/low-dose STZ (35 mg/kg) -T2D rats (manuscript in preparation) (Aleali et al., 2023). Notably, 4 days of sustained ZT-01 infusion significantly raised the blood glucose nadir during insulin-induced hypoglycemia without affecting oral glucose tolerance or basal glucagon levels (Aleali et al., 2023). This pilot study suggests that sustained delivery of SSTR2a by infusion pump therapy may be another viable approach for longer-term drug dosing.

**FIGURE 4:**
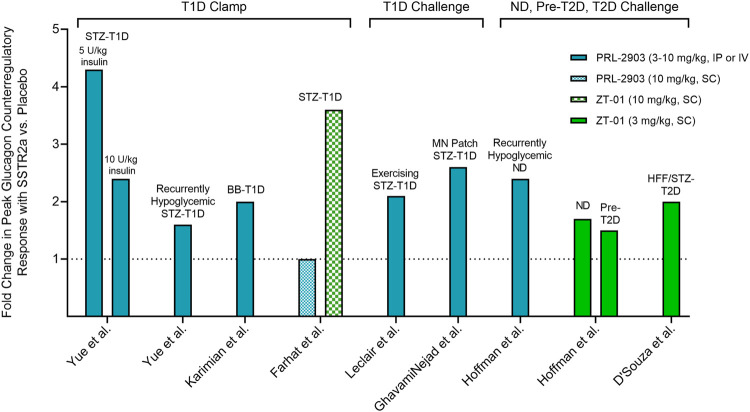
Fold change in the peak plasma glucagon response to insulin-induced hypoglycemia in rodents pre-treated with SSTR2a *versus* placebo. Fold change in glucagon represents peak glucagon concentration during level 2 hypoglycemia (<3.0 mmol/L) with SSTR2a divided by peak glucagon concentration with placebo. Rodent models of T1D showed a ∼2.5-fold improvement in peak glucagon response, on average, with SSTR2a *versus* placebo, whereas animal models with more intact counterregulation (i.e., non-diabetic and pre-T2D) were less responsive to treatment (see text). Clamp: hypoglycemia clamp; Challenge: bolus insulin challenge. STZ: streptozotocin; T1D: type 1 diabetes; BB: biobreeding; MN: microneedle; ND: non-diabetic; Pre-T2D: pre-type 2 diabetic; HFF: high-fat-fed; IP: intraperitoneal; IV: intravenous; SC: subcutaneous.

Figure 4 summarizes the fold-change in peak glucagon response to SSTR2a *versus* placebo during insulin-induced hypoglycemia in non-clinical studies to date. Rodent models of T1D showed a ∼2.5-fold improvement in peak glucagon response, on average, with SSTR2a *versus* placebo, whereas animal models with more intact counterregulation (i.e., non-diabetic and pre-diabetic) were less responsive to treatment. We chose to represent the glucagon response by peak values rather than the change from baseline to peak values, since baseline values were not available in all studies and peak values alone proved less sensitive to differences in methods of glucagon detection, which have become more selective over time. The observed variability in responses to drug treatment may reflect methodological differences relating to animal model, formulation and/or dose of SSTR2a, dose of insulin used to induce hypoglycemia, and route of administered test substances. Of particular note, PRL-2903 augmented peak glucagon levels when administered intravenously or intraperitoneally but not subcutaneously. Moreover, these data represent relative differences in systemic glucagon concentration with SSTR2a *versus* placebo and not absolute changes in glucagon concentration, which way be misleading since hepatic glucose production is largely determined by the ratio of insulin to glucagon in the portal vein (Sindelar et al., 1998).

## 6 Beyond hypoglycemia prevention: Therapeutic applications of SSTRa in diabetes management

Beyond hypoglycemia prevention, SSTRa’s may be capable of improving whole-body glucose metabolism through an incretin-dependent mechanism. Glucagon-like peptide-1 (GLP-1) is an incretin hormone secreted by enteroendocrine L-cells of the small intestine. GLP-1 reduces postprandial glucose excursions by stimulating insulin release from pancreatic β-cells, inhibiting gastric motility, and promoting satiety (Baggio and Drucker, 2007). People with obesity and/or T2D have impaired incretin release (Toft-Nielsen et al., 2001; Vilsbøll et al., 2001) and/or function (Nauck et al., 1986; Muscelli et al., 2008), and GLP-1-targeted therapies offer effective management of body weight and blood glucose levels, with underlying improvements to β-cell function and peripheral insulin sensitivity (Zander et al., 2002; Hinnen, 2017). It is well established that gut-derived SST inhibits GLP-1 secretion by paracrine effect, mediated primarily by SSTR5 on intestinal L-cells (Jepsen et al., 2019). Several SSTR5 antagonists (SSTR5a) have been developed in the past decade, and their therapeutic effects on glucose intolerance have been demonstrated in healthy mice (Liu et al., 2018; Jepsen et al., 2021), Zucker diabetic fatty (ZDF) rats (Sprecher et al., 2010; Farb et al., 2017), diet-induced obese (DIO) mice (Sprecher et al., 2010; Farb et al., 2017; Jepsen et al., 2021), and KK-Ay/Ta Jcl (KK-Ay) mice a model of obese T2D with severe insulin resistance (Tamura et al., 2023). Coadministration of a SSTR5a and a dipeptidyl peptidase IV inhibitor (DPP-4i) exerted synergistic effects on glucose tolerance, as well as circulating levels of insulin and active GLP-1 during an OGTT in healthy (Liu et al., 2018), ZDF (Farb et al., 2017), and DIO (Farb et al., 2017) rodents. The risk of hypoglycemia associated with SSTR5 antagonism was determined to be low in 4 h fasted lean C57BL/6N mice, since a supra-efficacious dose (30 mg/kg) of SSTR5a did not reduce basal glucose levels for 5 h after administration (Liu et al., 2018).

*In vivo* findings from male and female mice without diabetes suggest that treatment with SSTR2a and/or SSTR5a may lower blood glucose levels during an OGTT by mechanisms that are partially (via SSTR2a) or entirely (via SSTR5a) dependent on the stimulation of intestinally-derived GLP-1 (Jepsen et al., 2021). Since SSTR2a also stimulates insulin secretion by a direct effect on islet β-cells, engaging these complementary pathways through combined SSTR2 and SSTR5 antagonism may have additive effects on insulin release and whole-body glucose metabolism.

Two-week oral administration of an SSTR5a has been shown to significantly increase insulin sensitivity in KK-Ay mice, independent of weight loss or changes to food intake. While the mechanism is unclear, SST may inhibit hepatic insulin signaling by blocking insulin-induced AKT phosphorylation, an effect that may be reversible with SSTR5a in mice. Since hepatic insulin resistance can impair glucagon signaling and gluconeogenesis, restoring hepatic insulin sensitivity may have indirect benefits for glucose counterregulation (Tamura et al., 2023).

## 7 Summary and future directions

Diabetes is characterized as a bi-hormonal disorder, owing to the defective secretion of both glucoregulatory hormones, insulin and glucagon. This dysregulation can result in frequent exposure to hypoglycemia once treatment with insulin and/or insulin secretagogues is initiated. Hypoglycemia often becomes the major barrier to optimal glycemic management for individuals living with T1D or the more advanced stages of T2D. While the pathophysiology of defective glucagon counterregulation during treatment-induced hypoglycemia in T1D and advanced T2D remains unclear, mounting evidence suggests that the hypersecretion of inhibitory paracrine hormone, SST, may suppress counterregulatory glucagon secretion via SSTR2 expressed by α-cells. Selective antagonism of SSTR2 has been shown in various non-clinical models of T1D and in early phase clinical trials to at least partially restore glucagon counterregulation, with the goal of reducing hypoglycemia exposure in insulin-requiring diabetes. Future studies will help clarify the therapeutic potential of this new drug class in eliminating the burden of treatment-induced hypoglycemia and improving overall metabolic control in diabetes.

## References

[B1] Abdel-Halim S. M. Guenifi A. Efendić S. Ostenson C. G. (1993). Both somatostatin and insulin responses to glucose are impaired in the perfused pancreas of the spontaneously noninsulin-dependent diabetic GK (Goto-Kakizaki) rats. Acta Physiol. Scand. 148 (2), 219–226. 10.1111/j.1748-1716.1993.tb09551.x 8102504

[B2] Abitbol A. Peers S. Simonson E. Midmer M. Riddell M. Liggins R. (2023). 219-OR: Glucagon counterregulation in a hypoglycemic clamp in type 1 diabetes Is increased by ZT-01, a novel somatostatin receptor 2 antagonist—a phase 1b study. Diabetes 72 (1), 72. 10.2337/db23-219-or

[B3] Adam J. Ramracheya R. Chibalina M. V. Ternette N. Hamilton A. Tarasov A. I. (2017). Fumarate hydratase deletion in pancreatic β cells leads to progressive diabetes. Cell Rep. 20 (13), 3135–3148. 10.1016/j.celrep.2017.08.093 28954230 PMC5637167

[B4] Adapp C. (2022). 6. Glycemic targets: standards of medical care in diabetes-2022. Diabetes Care 45 (1), S83–S96. 10.2337/dc22-S006 34964868

[B79] ADVANCE Collaborative Group Patel A. MacMahon S. Chalmers J. Neal B. Billot L. (2008). Intensive blood glucose control and vascular outcomes in patients with type 2 diabetes. N. Engl. J. Med. 358 (24), 2560–2572. 10.1056/NEJMoa0802987 18539916

[B5] Aleali N. D’Souza N. C. Shakeri D. Simonson E. Liggins R. Chan O. (2023). 3-LB: Effects of sustained somatostatin receptor 2 antagonism (SSTR2a) on glycemia in type 2 diabetic (T2D) male rats—a pilot study. Diabetes 72 (1). 10.2337/db23-3-lb

[B6] Aronson R. Goldenberg R. Boras D. Skovgaard R. Bajaj H. (2018). The Canadian hypoglycemia assessment tool program: insights into rates and implications of hypoglycemia from an observational study. Can. J. Diabetes 42 (1), 11–17. 10.1016/j.jcjd.2017.01.007 28528246

[B7] Baetens D. Stefan Y. Ravazzola M. Malaisse-Lagae F. Coleman D. L. Orci L. (1978). Alteration of islet cell populations in spontaneously diabetic mice. Diabetes 27 (1), 1–7. 10.2337/diab.27.1.1 340309

[B8] Baggio L. L. Drucker D. J. (2007). Biology of incretins: GLP-1 and GIP. Gastroenterology 132 (6), 2131–2157. 10.1053/j.gastro.2007.03.054 17498508

[B9] Beck R. W. Bergenstal R. M. Riddlesworth T. D. Kollman C. (2019). The association of biochemical hypoglycemia with the subsequent risk of a Severe hypoglycemic event: analysis of the DCCT data set. Diabetes Technol. Ther. 21 (1), 1–5. 10.1089/dia.2018.0362 30575408 PMC6909677

[B10] Berlin I. Sachon C. Grimaldi A. (2005). Identification of factors associated with impaired hypoglycaemia awareness in patients with type 1 and type 2 diabetes mellitus. Diabetes Metab. 31 (3), 246–251. 10.1016/s1262-3636(07)70191-x 16142015

[B11] Berts A. Ball A. Dryselius S. Gylfe E. Hellman B. (1996). Glucose stimulation of somatostatin-producing islet cells involves oscillatory Ca2+ signaling. Endocrinology 137 (2), 693–697. 10.1210/endo.137.2.8593819 8593819

[B12] Bischof M. G. Bernroider E. Krssak M. Krebs M. Stingl H. Nowotny P. (2002). Hepatic glycogen metabolism in type 1 diabetes after long-term near normoglycemia. Diabetes 51 (1), 49–54. 10.2337/diabetes.51.1.49 11756322

[B13] Braun M. (2014). The somatostatin receptor in human pancreatic β-CellsVitamins Hormones. Academic Press, 165–193. 10.1016/B978-0-12-800174-5.00007-7 24559918

[B14] Braun M. Ramracheya R. Bengtsson M. Clark A. Walker J. N. Johnson P. R. (2010). Gamma-aminobutyric acid (GABA) is an autocrine excitatory transmitter in human pancreatic beta-cells. Diabetes 59 (7), 1694–1701. 10.2337/db09-0797 20413510 PMC2889769

[B15] Briant L. Salehi A. Vergari E. Zhang Q. Rorsman P. (2016). Glucagon secretion from pancreatic α-cells. Ups. J. Med. Sci. 121 (2), 113–119. 10.3109/03009734.2016.1156789 27044683 PMC4900066

[B16] Briant L. J. B. Reinbothe T. M. Spiliotis I. Miranda C. Rodriguez B. Rorsman P. (2018). δ-cells and β-cells are electrically coupled and regulate α-cell activity via somatostatin. J. Physiol. 596 (2), 197–215. 10.1113/JP274581 28975620 PMC5767697

[B17] Chow E. Bernjak A. Williams S. Fawdry R. A. Hibbert S. Freeman J. (2014). Risk of cardiac arrhythmias during hypoglycemia in patients with type 2 diabetes and cardiovascular risk. Diabetes 63 (5), 1738–1747. 10.2337/db13-0468 24757202

[B18] Cryer P. (2002). Hypoglycaemia: the limiting factor in the glycaemic management of type I and type II diabetes. Diabetologia 45 (7), 937–948. 10.1007/s00125-002-0822-9 12136392

[B19] Cryer P. E. (2005). Mechanisms of hypoglycemia-associated autonomic failure and its component syndromes in diabetes. Diabetes 54 (12), 3592–3601. 10.2337/diabetes.54.12.3592 16306382

[B20] Cryer P. E. Axelrod L. Grossman A. B. Heller S. R. Montori V. M. Seaquist E. R. (2009). Evaluation and management of adult hypoglycemic disorders: an endocrine society clinical practice guideline. J. Clin. Endocrinol. Metabolism 94 (3), 709–728. 10.1210/jc.2008-1410 19088155

[B21] Cryer P. E. Tse T. F. Clutter W. E. Shah S. D. (1984). Roles of glucagon and epinephrine in hypoglycemic and nonhypoglycemic glucose counterregulation in humans. Am. J. Physiol. 247 (2 Pt 1), E198–E205. 10.1152/ajpendo.1984.247.2.E198 6147094

[B22] Davis S. N. Mann S. Briscoe V. J. Ertl A. C. Tate D. B. (2009). Effects of intensive therapy and antecedent hypoglycemia on counterregulatory responses to hypoglycemia in type 2 diabetes. Diabetes 58 (3), 701–709. 10.2337/db08-1230 19073776 PMC2646069

[B23] de Heer J. Rasmussen C. Coy D. H. Holst J. J. (2008). Glucagon-like peptide-1, but not glucose-dependent insulinotropic peptide, inhibits glucagon secretion via somatostatin (receptor subtype 2) in the perfused rat pancreas. Diabetologia 51 (12), 2263–2270. 10.1007/s00125-008-1149-y 18795252

[B24] Dickerson M. T. Dadi P. K. Zaborska K. E. Nakhe A. Y. Schaub C. M. Dobson J. R. (2022). Gi/o protein-coupled receptor inhibition of beta-cell electrical excitability and insulin secretion depends on Na+/K+ ATPase activation. Nat. Commun. 13 (1), 6461. 10.1038/s41467-022-34166-z 36309517 PMC9617941

[B25] D’Souza N. C. Aiken J. Hoffman E. G. Atherley S. C. Champsi S. Aleali N. (2023a). 1-LB: Evaluating the effectiveness of the novel somatostatin receptor antagonist (SSTR2a) ZT-01 for hypoglycemia prevention in a rodent model of type 2 diabetes. Diabetes 72 (1). 10.2337/db23-1-lb PMC10951717 38510652

[B26] D’Souza N. C. Aleali N. Shakeri D. Hoffman E. G. Atherley S. C. Champsi S. (2023b). 2-LB: Effect of somatostatin receptor 2 antagonism (SSTR2a) on oral glucose tolerance (OGT) in a rat model of type 2 diabetes (T2D). Diabetes 72 (1), 72. 10.2337/db23-2-lb

[B27] Duckworth W. Abraira C. Moritz T. Reda D. Emanuele N. Reaven P. D. (2009). Glucose control and vascular complications in veterans with type 2 diabetes. N. Engl. J. Med. 360 (2), 129–139. 10.1056/NEJMoa0808431 19092145

[B28] Elliott A. D. Ustione A. Piston D. W. (2015). Somatostatin and insulin mediate glucose-inhibited glucagon secretion in the pancreatic α-cell by lowering cAMP. Am. J. Physiol. Endocrinol. Metab. 308 (2), E130–E143. 10.1152/ajpendo.00344.2014 25406263 PMC4297778

[B29] Farb T. B. Adeva M. Beauchamp T. J. Cabrera O. Coates D. A. Meredith T. D. (2017). Regulation of endogenous (male) rodent GLP-1 secretion and human islet insulin secretion by antagonism of somatostatin receptor 5. Endocrinology 158 (11), 3859–3873. 10.1210/en.2017-00639 28938487

[B30] Farhat R. Aiken J. D’Souza N. C. Appadurai D. Hull G. Simonson E. (2022). ZT-01: a novel somatostatin receptor 2 antagonist for restoring the glucagon response to hypoglycaemia in type 1 diabetes. Diabetes Obes. Metab. 24 (5), 908–917. 10.1111/dom.14652 35060297

[B31] Flatt A. J. Peleckis A. J. Dalton-Bakes C. Nguyen H. L. Ilany S. Matus A. (2023). Automated insulin delivery for hypoglycemia avoidance and glucose counterregulation in long-standing type 1 diabetes with hypoglycemia unawareness. Diabetes Technol. Ther. 25 (5), 302–314. 10.1089/dia.2022.0506 36763336 PMC10171955

[B32] Folli F. La Rosa S. Finzi G. Davalli A. M. Galli A. Dick E. J. (2018). Pancreatic islet of Langerhans’ cytoarchitecture and ultrastructure in normal glucose tolerance and in type 2 diabetes mellitus. Diabetes Obes. Metab. 20 (Suppl. 2), 137–144. 10.1111/dom.13380 30230173

[B33] Francis B. H. Baskin D. G. Saunders D. R. Ensinck J. W. (1990). Distribution of somatostatin-14 and somatostatin-28 gastrointestinal-pancreatic cells of rats and humans. Gastroenterology 99 (5), 1283–1291. 10.1016/0016-5085(90)91151-u 1976560

[B34] Gaisano H. Y. MacDonald P. E. Vranic M. (2012). Glucagon secretion and signaling in the development of diabetes. Front. Physiol. 3, 349. 10.3389/fphys.2012.00349 22969729 PMC3432929

[B35] GhavamiNejad A. Lu B. Samarikhalaj M. Liu J. F. Mirzaie S. Pereira S. (2022). Transdermal delivery of a somatostatin receptor type 2 antagonist using microneedle patch technology for hypoglycemia prevention. Drug Deliv. Transl. Res. 12 (4), 792–804. 10.1007/s13346-021-00944-3 33683625

[B36] Gilon P. (2020). The role of α-cells in islet function and glucose homeostasis in health and type 2 diabetes. J. Mol. Biol. 432 (5), 1367–1394. 10.1016/j.jmb.2020.01.004 31954131

[B37] Gregg E. W. (2017). The changing tides of the type 2 diabetes epidemic-smooth sailing or troubled waters ahead? Kelly West Award Lecture 2016. Diabetes Care 40 (10), 1289–1297. 10.2337/dci16-0055 28798086

[B38] Gutniak M. Grill V. Wiechel K. L. Efendić S. (1987). Basal and meal-induced somatostatin-like immunoreactivity in healthy subjects and in IDDM and totally pancreatectomized patients. Effects of acute blood glucose normalization. Diabetes 36 (7), 802–807. 10.2337/diab.36.7.802 2884157

[B39] Hartig S. M. Cox A. R. (2020). Paracrine signaling in islet function and survival. J. Mol. Med. 98 (4), 451–467. 10.1007/s00109-020-01887-x 32067063 PMC7899133

[B40] Hauge-Evans A. C. King A. J. Carmignac D. Richardson C. C. Robinson I. C. A. F. Low M. J. (2009). Somatostatin secreted by islet δ-cells fulfills multiple roles as a paracrine regulator of islet function. Diabetes 58 (2), 403–411. 10.2337/db08-0792 18984743 PMC2628614

[B41] Heinemann L. Freckmann G. Ehrmann D. Faber-Heinemann G. Guerra S. Waldenmaier D. (2018). Real-time continuous glucose monitoring in adults with type 1 diabetes and impaired hypoglycaemia awareness or severe hypoglycaemia treated with multiple daily insulin injections (HypoDE): a multicentre, randomised controlled trial. Lancet 391 (10128), 1367–1377. 10.1016/S0140-6736(18)30297-6 29459019

[B42] Heller S. R. Choudhary P. Davies C. Emery C. Campbell M. J. Freeman J. (2007). Risk of hypoglycaemia in types 1 and 2 diabetes: effects of treatment modalities and their duration. Diabetologia 50 (6), 1140–1147. 10.1007/s00125-007-0599-y 17415551

[B43] Heller S. R. Cryer P. E. (1991). Reduced neuroendocrine and symptomatic responses to subsequent hypoglycemia after 1 episode of hypoglycemia in nondiabetic humans. Diabetes 40 (2), 223–226. 10.2337/diab.40.2.223 1991573

[B44] Hermansen K. (1981). Characterisation of the abnormal pancreatic D and A cell function in streptozotocin diabetic dogs: studies with D-glyceraldehyde, dihydroxyacetone, D-mannoheptulose, D-glucose, and L-arginine. Diabetologia 21, 94. 10.1007/bf00257791 6117495

[B45] Hermansen K. Orskov H. Christensen S. E. (1979). Streptozotocin diabetes: a glucoreceptor dysfunction affecting D cells as well as B and A cells. Diabetologia 17 (6), 385–389. 10.1007/BF01236274 395006

[B46] Hinnen D. (2017). Glucagon-like peptide 1 receptor agonists for type 2 diabetes. Diabetes Spectr. 30 (3), 202–210. 10.2337/ds16-0026 28848315 PMC5556578

[B47] Hoffman E. G. D’Souza N. C. Aiken J. Atherley S. Liggins R. Riddell M. C. (2023). Effects of somatostatin receptor type 2 antagonism during insulin-induced hypoglycaemia in male rats with prediabetes. Diabetes Obes. Metab. 25 (6), 1547–1556. 10.1111/dom.15002 36734462

[B48] Hoffman E. G. Jahangiriesmaili M. Mandel E. R. Greenberg C. Aiken J. D'Souza N. C. (2021). Somatostatin receptor antagonism reverses glucagon counterregulatory failure in recurrently hypoglycemic male rats. Endocrinology 162 (12), bqab189. 10.1210/endocr/bqab189 34477204 PMC8482965

[B49] Hsu W. H. Xiang H. D. Rajan A. S. Kunze D. L. Boyd A. E. (1991). Somatostatin inhibits insulin secretion by a G-protein-mediated decrease in Ca2+ entry through voltage-dependent Ca2+ channels in the beta cell. J. Biol. Chem. 266 (2), 837–843. 10.1016/s0021-9258(17)35249-3 1702440

[B50] Huising M. O. van der Meulen T. Huang J. L. Pourhosseinzadeh M. S. Noguchi G. M. (2018). The difference δ-cells make in glucose control. Physiology 33 (6), 403–411. 10.1152/physiol.00029.2018 30303773 PMC6347098

[B51] International Hypoglycaemia Study Group (2017). Glucose concentrations of less than 3.0 mmol/L (54 mg/dL) should be reported in clinical trials: a joint position statement of the American Diabetes Association and the European Association for the Study of Diabetes. Diabetes Care 40 (1), 155–157. 10.2337/dc16-2215 27872155

[B52] Jepsen S. L. Grunddal K. V. Wewer Albrechtsen N. J. Engelstoft M. S. Gabe M. B. N. Jensen E. P. (2019). Paracrine crosstalk between intestinal L- and D-cells controls secretion of glucagon-like peptide-1 in mice. Am. J. Physiology-Endocrinology Metabolism 317 (6), E1081–E1093. 10.1152/ajpendo.00239.2019 PMC6962500 31503512

[B53] Jepsen S. L. Wewer Albrechtsen N. J. Windeløv J. A. Galsgaard K. D. Hunt J. E. Farb T. B. (2021). Antagonizing somatostatin receptor subtype 2 and 5 reduces blood glucose in a gut- and GLP-1R-dependent manner. JCI Insight 6 (4), e143228. 10.1172/jci.insight.143228 33434183 PMC7934931

[B54] Kailey B. van de Bunt M. Cheley S. Johnson P. R. MacDonald P. E. Gloyn A. L. (2012). SSTR2 is the functionally dominant somatostatin receptor in human pancreatic β- and α-cells. Am. J. Physiology-Endocrinology Metabolism 303 (9), E1107–E1116. 10.1152/ajpendo.00207.2012 PMC3492856 22932785

[B55] Karimian N. Qin T. Liang T. Osundiji M. Huang Y. Teich T. (2013). Somatostatin receptor type 2 antagonism improves glucagon counterregulation in biobreeding diabetic rats. Diabetes 62 (8), 2968–2977. 10.2337/db13-0164 23630299 PMC3717832

[B56] Kellard J. A. Rorsman N. J. G. Hill T. G. Armour S. L. van de Bunt M. Rorsman P. (2020). Reduced somatostatin signalling leads to hypersecretion of glucagon in mice fed a high-fat diet. Mol. Metab. 40, 101021. 10.1016/j.molmet.2020.101021 32446876 PMC7322681

[B57] Kumar U. Sasi R. Suresh S. Patel A. Thangaraju M. Metrakos P. (1999). Subtype-selective expression of the five somatostatin receptors (hSSTR1-5) in human pancreatic islet cells: a quantitative double-label immunohistochemical analysis. Diabetes 48 (1), 77–85. 10.2337/diabetes.48.1.77 9892225

[B58] Lane W. Bailey T. S. Gerety G. Gumprecht J. Philis-Tsimikas A. Hansen C. T. (2017). Effect of insulin degludec vs insulin glargine U100 on hypoglycemia in patients with type 1 diabetes: the SWITCH 1 randomized clinical trial. JAMA 318 (1), 33–44. 10.1001/jama.2017.7115 28672316 PMC5817477

[B59] Leclair E. Liggins R. T. Peckett A. J. Teich T. Coy D. H. Vranic M. (2016). Glucagon responses to exercise-induced hypoglycaemia are improved by somatostatin receptor type 2 antagonism in a rat model of diabetes. Diabetologia 59 (8), 1724–1731. 10.1007/s00125-016-3953-0 27075449

[B60] Lin Y. K. Hung M. Sharma A. Chan O. Varner M. W. Staskus G. (2019). Impaired awareness of hypoglycemia continues to be a risk factor for severe hypoglycemia despite the use of continuous glucose monitoring system in type 1 diabetes. Endocr. Pract. 25 (6), 517–525. 10.4158/EP-2018-0527 30865520 PMC6771275

[B61] Lipska K. J. Ross J. S. Wang Y. Inzucchi S. E. Minges K. Karter A. J. (2014). National trends in US hospital admissions for hyperglycemia and hypoglycemia among Medicare beneficiaries, 1999 to 2011. JAMA Intern Med. 174 (7), 1116–1124. 10.1001/jamainternmed.2014.1824 24838229 PMC4152370

[B62] Lipska K. J. Yao X. Herrin J. McCoy R. G. Ross J. S. Steinman M. A. (2017). Trends in drug utilization, glycemic control, and rates of severe hypoglycemia, 2006-2013. Diabetes Care 40 (4), 468–475. 10.2337/dc16-0985 27659408 PMC5360291

[B63] Liu D. McManus R. M. Ryan E. A. (1996). Improved counter-regulatory hormonal and symptomatic responses to hypoglycemia in patients with insulin-dependent diabetes mellitus after 3 months of less strict glycemic control. Clin. Invest Med. 19 (2), 71–82 . http://www.ncbi.nlm.nih.gov/pubmed/8697673 (Accessed November 23, 2018). 8697673

[B64] Liu W. Shao P. P. Liang G. B. Bawiec J. He J. Aster S. D. (2018). Discovery and pharmacology of a novel somatostatin subtype 5 (SSTR5) antagonist: synergy with DPP-4 inhibition. ACS Med. Chem. Lett. 9 (11), 1082–1087. 10.1021/acsmedchemlett.8b00305 30429949 PMC6231191

[B65] MacDonald P. E. De Marinis Y. Z. Ramracheya R. Salehi A. Ma X. Johnson P. R. (2007). “A K ATP channel-dependent pathway within alpha cells regulates glucagon release from both rodent and human islets of Langerhans, PLoS Biol. 5, e143. 10.1371/journal.pbio.0050143 17503968 PMC1868042

[B66] Madsbad S. Hilsted J. Krarup T. Sestoft L. Christensen N. J. Faber O. K. (1982). Hormonal, metabolic and cardiovascular responses to hypoglycaemia in type 1 (insulin-dependent) diabetes with and without residual B cell function. Diabetologia 23, 499–503. 10.1007/BF00254298 6759276

[B67] Mallad A. Hinshaw L. Schiavon M. Dalla Man C. Dadlani V. Basu R. (2015). Exercise effects on postprandial glucose metabolism in type 1 diabetes: a triple-tracer approach. Am. J. Physiology-Endocrinology Metabolism 308 (12), E1106–E1115. 10.1152/ajpendo.00014.2015 PMC4469811 25898950

[B68] Marre M. Miller J. Helman A. M. Assan R. (1983). Reciprocal gastropancreatic modulations for the release of somatostatin-like immunoreactivity, glucagon, and insulin in the rat. Diabetes 32 (8), 768–773. 10.2337/diab.32.8.768 6347773

[B69] McCarthy O. Schmidt S. Christensen M. B. Bain S. C. Nørgaard K. Bracken R. (2022). The endocrine pancreas during exercise in people with and without type 1 diabetes: beyond the beta-cell. Front. Endocrinol. (Lausanne) 13, 981723. 10.3389/fendo.2022.981723 36147573 PMC9485437

[B70] McCoy R. G. Van Houten H. K. Ziegenfuss J. Y. Shah N. D. Wermers R. A. Smith S. A. (2012). Increased mortality of patients with diabetes reporting severe hypoglycemia. Diabetes Care 35 (9), 1897–1901. 10.2337/dc11-2054 22699297 PMC3425008

[B71] Miranda C. Begum M. Vergari E. Briant L. J. B. (2022). Gap junction coupling and islet delta-cell function in health and disease. Pept. (NY) 147, 170704. 10.1016/j.peptides.2021.170704 34826505

[B72] Mitrakou A. Ryan C. Veneman T. Mokan M. Jenssen T. Kiss I. (1991). Hierarchy of glycemic thresholds for counterregulatory hormone secretion, symptoms, and cerebral dysfunction. Am. J. Physiology-Endocrinology Metabolism 260 (1), E67–E74. 10.1152/ajpendo.1991.260.1.E67 1987794

[B73] Muscelli E. Mari A. Casolaro A. Camastra S. Seghieri G. Gastaldelli A. (2008). Separate impact of obesity and glucose tolerance on the incretin effect in normal subjects and type 2 diabetic patients. Diabetes 57 (5), 1340–1348. 10.2337/db07-1315 18162504

[B74] Nauck M. Stöckmann F. Ebert R. Creutzfeldt W. (1986). Reduced incretin effect in type 2 (non-insulin-dependent) diabetes. Diabetologia 29 (1), 46–52. 10.1007/BF02427280 3514343

[B117] NCT05007977 (2023). Effect of ZT-01 on glucagon during hypoglycemia in type 1 diabetes mellitus. Identifier: NCT05007977 https://www.clinicaltrials.gov/ct2/show/NCT05007977.

[B118] NCT05762107 (2023). A study of the effect of ZT-01 on night-time hypoglycemia in type 1 diabetes (ZONE). Identifier: NCT05762107 https://clinicaltrials.gov/study/NCT05762107.

[B75] Noguchi G. M. Huising M. O. (2019). Integrating the inputs that shape pancreatic islet hormone release. Nat. Metab. 1 (12), 1189–1201. 10.1038/s42255-019-0148-2 32694675 PMC7378277

[B76] Omar-Hmeadi M. Lund P. E. Gandasi N. R. Tengholm A. Barg S. (2020). Paracrine control of α-cell glucagon exocytosis is compromised in human type-2 diabetes. Nat. Commun. 11 (1), 1896. 10.1038/s41467-020-15717-8 32312960 PMC7171169

[B77] Orci L. Baetens D. Rufener C. Amherdt M. Ravazzola M. Studer P. (1976). Hypertrophy and hyperplasia of somatostatin-containing D-cells in diabetes. Proc. Natl. Acad. Sci. 73 (4), 1338–1342. 10.1073/pnas.73.4.1338 131313 PMC430269

[B78] Patel A. Chalmers J. Poulter N. (2005). ADVANCE: action in diabetes and vascular disease. J. Hum. Hypertens. 19 (S1), S27–S32. 10.1038/sj.jhh.1001890 16075030

[B80] Peacey S. R. Robinson R. Bedford C. Harris N. D. Macdonald I. A. Holman R. R. (2000). Does the choice of treatment for type 2 diabetes affect the physiological response to hypoglycemia? Diabetes Care 23 (7), 1022–1023. 10.2337/diacare.23.7.1022 10895860

[B81] Rahier J. Goebbels R. M. Henquin J. C. (1983). Cellular composition of the human diabetic pancreas. Diabetologia 24 (5), 366–371. 10.1007/BF00251826 6347784

[B82] Ramracheya R. Ward C. Shigeto M. Walker J. N. Amisten S. Zhang Q. (2010). Membrane potential-dependent inactivation of voltage-gated ion channels in alpha-cells inhibits glucagon secretion from human islets. Diabetes 59 (9), 2198–2208. 10.2337/db09-1505 20547976 PMC2927942

[B83] Ratner R. E. (2018). Hypoglycemia: new definitions and regulatory implications. Diabetes Technol. Ther. 20 (S2), S250–S253. 10.1089/dia.2018.0113 29873525

[B84] Raulf F. Pérez J. Hoyer D. Bruns C. (1994). Differential expression of five somatostatin receptor subtypes, SSTR1-5, in the CNS and peripheral tissue. Digestion 55 (3), 46–53. 10.1159/000201201 7698537

[B85] Rickels M. R. (2019). Hypoglycemia-associated autonomic failure, counterregulatory responses, and therapeutic options in type 1 diabetes. Ann. N. Y. Acad. Sci. 1454 (1), 68–79. 10.1111/nyas.14214 31389033 PMC6945804

[B86] Rorsman P. Ashcroft F. M. (2018). Pancreatic β-Cell electrical activity and insulin secretion: of mice and men. Physiol. Rev. 98 (1), 117–214. 10.1152/physrev.00008.2017 29212789 PMC5866358

[B87] Rorsman P. Huising M. O. (2018). The somatostatin-secreting pancreatic δ-cell in health and disease. Nat. Rev. Endocrinol. 14 (7), 404–414. 10.1038/s41574-018-0020-6 29773871 PMC5997567

[B88] Segel S. A. Paramore D. S. Cryer P. E. (2002). Hypoglycemia-associated autonomic failure in advanced type 2 diabetes. Diabetes 51 (3), 724–733. 10.2337/diabetes.51.3.724 11872673

[B89] Siafarikas A. Johnston R. J. Bulsara M. K. O’Leary P. Jones T. W. Davis E. A. (2012). Early loss of the glucagon response to hypoglycemia in adolescents with type 1 diabetes. Diabetes Care 35 (8), 1757–1762. 10.2337/dc11-2010 22699295 PMC3402257

[B90] Sindelar D. K. Chu C. A. Venson P. Donahue E. P. Neal D. W. Cherrington A. D. (1998). Basal hepatic glucose production is regulated by the portal vein insulin concentration. Diabetes 47 (4), 523–529. 10.2337/diabetes.47.4.523 9568682

[B91] Singh B. Khattab F. Gilon P. (2022). Glucose inhibits glucagon secretion by decreasing [Ca2+]c and by reducing the efficacy of Ca2+ on exocytosis via somatostatin-dependent and independent mechanisms. Mol. Metab. 61, 101495. 10.1016/j.molmet.2022.101495 35421610 PMC9065434

[B92] Singh V. Brendel M. D. Zacharias S. Mergler S. Jahr H. Wiedenmann B. (2007). Characterization of somatostatin receptor subtype-specific regulation of insulin and glucagon secretion: an *in vitro* study on isolated human pancreatic islets. J. Clin. Endocrinol. Metab. 92 (2), 673–680. 10.1210/jc.2006-1578 17105845

[B93] Sprecher U. Mohr P. Martin R. E. Maerki H. P. Sanchez R. A. Binggeli A. (2010). Novel, non-peptidic somatostatin receptor subtype 5 antagonists improve glucose tolerance in rodents. Regul. Pept. 159 (1-3), 19–27. 10.1016/j.regpep.2009.09.006 19761802

[B94] Strowski M. Z. Parmar R. M. Blake A. D. Schaeffer J. M. (2000). Somatostatin inhibits insulin and glucagon secretion via two receptors subtypes: an *in vitro* study of pancreatic islets from somatostatin receptor 2 knockout mice. Endocrinology 141 (1), 111–117. 10.1210/endo.141.1.7263 10614629

[B95] Svendsen B. Holst J. J. (2021). Paracrine regulation of somatostatin secretion by insulin and glucagon in mouse pancreatic islets. Diabetologia 64 (1), 142–151. 10.1007/s00125-020-05288-0 33043402

[B96] Taborsky G. J. Ensinck J. W. (1984). Contribution of the pancreas to circulating somatostatin-like immunoreactivity in the normal dog. J. Clin. Investigation 73 (1), 216–223. 10.1172/JCI111194 PMC425002 6140271

[B97] Tamura Y. O. Sugama J. Abe S. Shimizu Y. Hirose H. Watanabe M. (2023). Selective somatostatin receptor 5 inhibition improves hepatic insulin sensitivity. Pharmacol. Res. Perspect. 11 (1), e01043. 10.1002/prp2.1043 36585794 PMC9803904

[B98] Toft-Nielsen M. B. Damholt M. B. Madsbad S. Hilsted L. M. Hughes T. E. Michelsen B. K. (2001). Determinants of the impaired secretion of glucagon-like peptide-1 in type 2 diabetic Patients. J. Clin. Endocrinol. Metab. 86 (8), 3717–3723. 10.1210/jcem.86.8.7750 11502801

[B99] van der Meulen T. Donaldson C. J. Cáceres E. Hunter A. E. Cowing-Zitron C. Pound L. D. (2015). Urocortin3 mediates somatostatin-dependent negative feedback control of insulin secretion. Nat. Med. 21 (7), 769–776. 10.1038/nm.3872 26076035 PMC4496282

[B100] Vergari E. Denwood G. Salehi A. Zhang Q. Adam J. Alrifaiy A. (2020). Somatostatin secretion by Na+-dependent Ca2+-induced Ca2+ release in pancreatic delta cells. Nat. Metab. 2 (1), 32–40. 10.1038/s42255-019-0158-0 31993555 PMC6986923

[B101] Vieira E. Salehi A. Gylfe E. (2007). Glucose inhibits glucagon secretion by a direct effect on mouse pancreatic alpha cells. Diabetologia 50 (2), 370–379. 10.1007/s00125-006-0511-1 17136393

[B102] Vilsbøll T. Krarup T. Deacon C. F. Madsbad S. Holst J. J. (2001). Reduced postprandial concentrations of intact biologically active glucagon-like peptide 1 in type 2 diabetic patients. Diabetes 50 (3), 609–613. 10.2337/diabetes.50.3.609 11246881

[B103] Walker J. N. Ramracheya R. Zhang Q. Johnson P. R. V. Braun M. Rorsman P. (2011). Regulation of glucagon secretion by glucose: paracrine, intrinsic or both? Diabetes Obes. Metab. 13 (s1), 95–105. 10.1111/j.1463-1326.2011.01450.x 21824262

[B104] Weinstock R. S. Xing D. Maahs D. M. Michels A. Rickels M. R. Peters A. L. (2013). Severe hypoglycemia and diabetic ketoacidosis in adults with type 1 diabetes: results from the T1D exchange clinic registry. J. Clin. Endocrinol. Metab. 98 (8), 3411–3419. 10.1210/jc.2013-1589 23760624

[B105] Weir G. C. Bonner-Weir S. (2023). Conflicting views about interactions between pancreatic α-cells and β-cells. Diabetes 72 (12), 1741–1747. 10.2337/db23-0292 37983524 PMC10658062

[B106] Weir G. C. Clore E. T. Zmachinski C. J. Bonner-Weir S. (1981). Islet secretion in a new experimental model for non-insulin-dependent diabetes. Diabetes 30 (7), 590–595. 10.2337/diab.30.7.590 6114005

[B107] Weir G. C. Laybutt D. R. Kaneto H. Bonner-Weir S. Sharma A. (2001). Beta-cell adaptation and decompensation during the progression of diabetes. Diabetes 50 (1), S154–S159. 10.2337/diabetes.50.2007.s154 11272180

[B108] Whitmer R. A. Karter A. J. Yaffe K. Quesenberry C. P. Selby J. V. (2009). Hypoglycemic episodes and risk of dementia in older patients with type 2 diabetes mellitus. JAMA 301 (15), 1565–1572. 10.1001/jama.2009.460 19366776 PMC2782622

[B109] Wysham C. Bhargava A. Chaykin L. de la Rosa R. Handelsman Y. Troelsen L. N. (2017). Effect of insulin degludec vs insulin glargine U100 on hypoglycemia in patients with type 2 diabetes: the SWITCH 2 randomized clinical trial. JAMA 318 (1), 45–56. 10.1001/jama.2017.7117 28672317 PMC5817473

[B110] Xu S. F. S. Andersen D. B. Izarzugaza J. M. G. Kuhre R. E. Holst J. J. (2020). In the rat pancreas, somatostatin tonically inhibits glucagon secretion and is required for glucose-induced inhibition of glucagon secretion. Acta Physiol. 229 (3), e13464. 10.1111/apha.13464 32145704

[B111] Yue J. T. Y. Burdett E. Coy D. H. Giacca A. Efendic S. Vranic M. (2012). Somatostatin receptor type 2 antagonism improves glucagon and corticosterone counterregulatory responses to hypoglycemia in streptozotocin-induced diabetic rats. Diabetes 61 (1), 197–207. 10.2337/db11-0690 22106159 PMC3237655

[B112] Yue J. T. Y. Riddell M. C. Burdett E. Coy D. H. Efendic S. Vranic M. (2013). Amelioration of hypoglycemia via somatostatin receptor type 2 antagonism in recurrently hypoglycemic diabetic rats. Diabetes 62 (7), 2215–2222. 10.2337/db12-1523 23434929 PMC3712070

[B113] Zammitt N. N. Frier B. M. (2005). Hypoglycemia in type 2 diabetes: pathophysiology, frequency, and effects of different treatment modalities. Diabetes Care 28 (12), 2948–2961. 10.2337/diacare.28.12.2948 16306561

[B114] Zander M. Madsbad S. Madsen J. L. Holst J. J. (2002). Effect of 6-week course of glucagon-like peptide 1 on glycaemic control, insulin sensitivity, and β-cell function in type 2 diabetes: a parallel-group study. Lancet 359 (9309), 824–830. 10.1016/S0140-6736(02)07952-7 11897280

[B115] Zenz S. Mader J. K. Regittnig W. Brunner M. Korsatko S. Boulgaropoulos B. (2018). Impact of C-Peptide status on the response of glucagon and endogenous glucose production to induced hypoglycemia in T1DM. J. Clin. Endocrinol. Metab. 103 (4), 1408–1417. 10.1210/jc.2017-01836 29408994

[B116] Zhong V. W. Juhaeri J. Cole S. R. Kontopantelis E. Shay C. M. Gordon-Larsen P. (2017). Incidence and trends in hypoglycemia hospitalization in adults with type 1 and type 2 diabetes in England, 1998–2013: a retrospective cohort study. Diabetes Care 40 (12), 1651–1660. 10.2337/dc16-2680 28716781

